# Parallel Offline Breath Sampling for Cross-Validated Analysis of Volatile Organic Compound Metabolites

**DOI:** 10.1007/s11306-025-02340-1

**Published:** 2025-09-17

**Authors:** Eray Schulz, Mariana Maciel, Zhige Wang, Shivaum Heranjal, Xiaowen Liu, Sha Cao, Ryan F. Relich, Mark Woollam, Mangilal Agarwal

**Affiliations:** 1 Chemistry & Chemical Biology, Indiana University Indianapolis, Indianapolis, IN USA; 2 Integrated Nanosystems Development Institute, Indiana University Indianapolis, Indianapolis, IN USA; 3 Department of Computer Science, Tulane University School of Engineering, New Orleans, LA USA; 4 Electrical & Computer Engineering, Purdue University Indianapolis, Indianapolis, IN USA; 5 Deming Department of Medicine, Tulane University School of Medicine, New Orleans, LA USA; 6 Biomedical Engineering, Oregon Health & Science University School of Medicine, Portland, OR USA; 7 Pathology & Laboratory Medicine, Indiana University School of Medicine, Indianapolis, IN USA; 8 Chemistry & Chemical Biology, School of Science, Indiana University Indianapolis, 402 N. Blackford Street, Indianapolis, IN 46202 USA; 9 Biomedical Engineering & Informatics, Luddy School of Informatics, Indiana University Indianapolis, Indianapolis, IN USA; 10 Biomedical Engineering & Informatics, Luddy School of Informatics of America, Indiana University Indianapolis, Indiana University, 535 W Michigan Street, Indianapolis, IN 46202 USA

**Keywords:** Volatile organic compounds (VOCs), Exhaled breath, Solid phase microextraction (SPME), Gas chromatography–mass spectrometry (GC-MS), Chemometric analysis

## Abstract

**Introduction:**

Volatile organic compounds (VOCs) in breath are potential biomarkers for medical conditions that may be used for non-invasive health monitoring. One challenge that still exists is determining the fidelity of reported VOC biomarkers. The lack of universally accepted sampling methods makes it difficult to identify reliable candidates, thus allowing for the potential of false discovery.

**Objectives:**

The purpose of this study was to robustly profile VOCs in breath samples collected from relatively healthy participants using two offline methods for collection/analysis via solid phase microextraction (SPME) coupled to gas chromatography – mass spectrometry (GC-MS).

**Methods:**

158 cross-sectional volunteers provided one-time samples using two methods, one which directly sampled breath via SPME and another which collected breath in Tedlar bags. Using both methods, 10 volunteers provided an additional nine longitudinal samples. Ambient air samples were collected routinely, and a robust data processing schematic was used to ensure high quality reporting of on-breath VOCs.

**Results:**

Data screening and processing led to the identification of > 30 unique VOCs in both methods. Hierarchical clustering and correlation analyses demonstrated volatile terpene/-oids showed homologous trends in both data sets. Of the 12 VOCs identified using both methods, 11 analytes displayed statistically significant correlations (*p* < 0.05) in healthy breath samples. Finally, both methods were benchmarked regarding VOC reproducibility, and analyses showed that longitudinally collected samples were more reproducible compared to cross-sectional.

**Conclusions:**

The quantitative results from both sampling methods mirrored each other, thus increasing the reliability and fidelity of VOCs reported along with the results from biostatistical analysis.

**Supplementary Information:**

The online version contains supplementary material available at 10.1007/s11306-025-02340-1.

## Introduction

The field of breathomics traces back to the fourth century, where Greek physician Hippocrates utilized odors within exhaled breath to help identify patients with medical conditions including liver disease, diabetes, and kidney failure (Sharma et al., 2023; Wallace & Pleil, 2018). In the early 1970 s, this field of research exploded after Nobel laureate Linus Pauling, the first scientist to utilize modern analytical methods to profile the chemical constituents of breath, identified 250 unique volatile organic compounds (VOCs) in samples analyzed by gas-liquid partition chromatography (Pauling et al., 1971). VOCs in exhaled breath are small molecules that originate from a variety of sources including inside and outside the human body. Endogenously produced VOCs are secondary metabolites and can be detected in a variety of biofluids, including but not limited to breath, urine, and feces (Amann et al., 2014; Feil et al., 2021; Phillips et al., 1999; Woollam et al., 2020). Among the viable sample types for VOC analysis, breath presents several advantages because it is highly non-invasive, of virtually limitless supply, and a rich source of potential biomarkers. The current gold standard for exhaled VOC analysis is gas chromatography-mass spectrometry (GC-MS) due to its ability to chromatographically resolve, structurally elucidate, and quantify volatile analytes in complex sample matrices. This technique is often coupled with preconcentration methods such as solid phase microextraction (SPME) or sorbent tubes due to the sparse nature of VOCs (Cai et al., 2006; Ge et al., 2022; Schulz et al., 2023). A variety of breath sampling methods exist, including polymer-based collection bags and commercially available devices designed for direct breath capture. The complexity increases as both SPME and sorbent tubes can be integrated across these different sampling approaches (Di Gilio et al., 2020). Many studies have demonstrated that there are unique biomarkers in exhaled breath for medical conditions including but not limited to diabetes (Dixit et al., 2021; Woollam et al., 2025a), lung cancer (Wang et al., 2022), COVID-19 (Woollam et al., 2022a), and heart failure (Yokokawa et al., 2017). Although progress has been made in biomarker discovery, historically there has been a limited understanding of the specific VOCs found in healthy breath.

Very recently there has been a resurgence to make strides in defining the chemical composition of breath samples collected from relatively healthy volunteers using standardized tools/methods. This is a particularly difficult task due to the lack of a gold standard method for breath sampling, as well as the need to ensure VOCs are not sourced from sampling materials or ambient air. Nonetheless, efforts are underway to investigate and develop a robust framework for confirming VOCs are on breath. For example, a study identified healthy breath VOCs in 90 participants using the Respiration Collector for In Vitro Analysis (ReCIVA) with thermal desorption coupled to GC-MS. The investigators implemented rigorous criteria to define VOCs on breath through comparison of background samples and running pure standards to verify identity. Criteria included breath VOC signals exceeding background by three standard deviations (in ≥ 50% of samples), statistically significant increases over background, and receiver operator characteristic area under the curve (ROC AUC) > 0.8 for distinguishing breath from background. This led to the detection of 1471 VOCs in > 80% of breath/background samples, with 148 analytes identified and validated to be enriched in exhaled breath (Arulvasan et al., 2024). Another study aimed to develop an average healthy human profile of molecules in exhaled breath using direct secondary ionization coupled with high resolution MS. Breath samples from 31 participants were collected which led to the identification of 48 unique compounds within the average healthy profile. The team also investigated correlations with confounding variables, identifying significant associations between breath-based data and sex, time-of-day, and even age (Sasiene et al., 2024). An important aspect of the previous study was the use of Metabolomics Standards Initiative (MSI) identification levels, which enhances confidence in reporting the results for on-breath VOC analysis (Sumner et al., 2007).

Although these previous studies robustly determine on-breath VOC profiles in a relatively healthy population, they use only a single method for breath sampling and analysis. In the same spirit to further understand VOC profiles and increase fidelity of analysis, our study recruited > 150 relatively healthy individuals who donated breath samples using two distinct SPME GC-MS methods which have been previously published. The first method utilized a direct breath sampling method termed DB-SPME (Schulz et al., 2023), and the second method used Tedlar breath collection bags with VOCs cryothermally transferred into headspace vials (Woollam et al., 2022b). In addition to cross-sectional analyses, both methods were also used to profile VOCs in a longitudinal cohort of 10 individuals providing a total of 10 samples over the course of six months. Herein, we demonstrate the benefits of utilizing two methods in parallel for quantitative analysis of exhaled breath VOCs in a relatively healthy population.

## Materials and methods

### Materials and instrumentation

ViroMax filters (A-M Systems, Sequim, WA, USA) were used to remove viral/bacterial agents during breath collection. A Philips NM3 capnograph (Murrysville, PA, USA) was used to monitor CO_2_ concentrations and exhalation volumes during the DB-SPME breath collection process. Glass Pasteur pipettes from Fisher Scientific (Hampton, NH, USA) and Tygon tubing from Saint-Gobain (Courbevoie, France) were used for DB-SPME. Three-liter Tedlar gas collection bags from Restek (Bellefonte, PA, USA) were also used for exhaled breath collection. An MCS-series mass flow controller acquired from Alicat Scientific (Marana, AZ, USA) was utilized for the cryothermal transfer of exhaled breath from Tedlar bags. Fourteen-gauge hypodermic stainless-steel needles (Med-Vet International, Mettawa, IL, USA), as well as deactivated glass wool and 20 mL headspace vials with magnetic screw-thread caps (Restek) were used to complete the cryothermal transfer process. An Agilent (Santa Clara, CA, USA) 7890 A GC coupled to a 7200 MS quadrupole time-of-flight (QTOF) system with a PAL autosampler (CTC Analytics; Raleigh, NC, USA) was used to measure VOCs in breath. Here, a Rxi-5ms GC column 30 m in length with a 0.25 mm internal diameter and 0.25 micrometer film thickness from Restek was implemented. Two-centimeter polydimethylsiloxane/carboxen/divinylbenzene (PDMS/CAR/DVB) SPME fibers from Supelco (Bellefonte, PA, USA) were used for VOC preconcentration. High density polyethylene (HDPE) pellets from The Fundamental Rockhound (Westminster, CO, USA) were used as an external standard to monitor instrumental drift daily. Pure VOC and internal standards were purchased from Sigma-Aldrich (St. Louis, MO, USA). These included but were not limited to deuterated acetone, α-pinene, β-pinene, *p*-cymene, limonene and menthol (see Supplementary Information for the full list of compounds analytically verified using standards). All materials described in this section are in Supplementary Table S.1 with their respective manufacturer part numbers.

### Volunteer recruitment

Participants were recruited through word of mouth as well as posters at the Indiana University-Purdue University Indianapolis (IUPUI) campus. Volunteers were recruited for cross-sectional sampling, with 10 individuals each providing 10 samples longitudinally over six months (≈ 21 days between samples). This study was approved by the IUPUI Institutional Review Board (IRB #s 12954 and 15542) along with the Institutional Biosafety Committee (protocol # IN-1301), and all volunteers provided written consent. All participants were healthy individuals between the ages of 18–80 and did not report serious underlying medical conditions. A lifestyle questionnaire was administered for confounding data (age, biological sex, body mass index (BMI), special/restricted diet, information on serious underlying medical conditions, chronic fatigue, symptoms of short-term illness, medications taken in the last 24 h, average hours of sleep, and smoking status), and blood samples were collected at each visit for comprehensive metabolic panel (CMP)/complete blood count (CBC) testing. A food diary was administered to collect information on food/drink intake the day before and day of sample collection. All volunteers avoided smoking, consuming any food/drink (other than water), and brushing their teeth at least one hour prior to sampling.

### Breath sampling protocols

A previously optimized breath sampling method termed DB-SPME was used to assess VOC profiles in exhaled breath (Supplementary Figure S.1(a). A detailed description of all methodological protocols and parameters for breath sampling using DB-SPME have been published previously (Schulz et al., 2023). Briefly, volunteers were instructed to exhale tidally through a viral filter which is connected to a glass pipette (using tubing) that houses the exposed SPME fiber. As volunteers exhale, VOCs travel through the system and are captured on the SPME fiber. Once 48 L of breath was sampled (confirmed via capnography), the SPME fiber was removed from the breath sampling apparatus, exposed to a 1 mL internal standard solution (deuterated acetone in water, 5ppm) for one minute, and injected manually into the GC-MS QTOF inlet for instrumental analysis. The entire sampling system was replaced with new materials prior to a new volunteer. Once the volunteer completed breath sampling with DB-SPME, they were provided with a pre-conditioned (equilibrated with ultra-high pure nitrogen for at least 3 min) 3 L Tedlar bag, where breath was collected for the cryothermal transfer method (Supplementary Figure S.1(b)). Volunteers were instructed to breathe tidally into the inlet of the bag until it was approximately 80% full. Immediately after collection, breath VOCs in the bag were transferred into a brand new 20 mL headspace vial (pre-conditioned at 70 °C for at least 24 h) loaded with glass wool over dry ice using a mass flow controller interfaced with a vacuum. Once breath was completely transferred from the bag, the headspace vial was sealed with Parafilm and stored in a −80 C° freezer until it was run for analysis. Additionally, ambient air samples were collected using the same apparatus/materials before the first volunteer and after the last volunteer every day of breath sampling to assess background levels of VOCs using both methods. Exhaled breath sampling parameters for both methodologies were previously optimized and detailed in published studies (Schulz et al., 2023; Siegel et al., 2017; Woollam et al., 2022a). All breath and background samples were collected in the same location, room and table over the course of the study.

### SPME GC-MS QTOF protocols

For cryothermally transferred samples, vials were thawed and extracted using the triple-phase SPME fiber for 45 min (60 °C and agitation at 250 RPM). Using both methods, once the VOCs were captured on the SPME fiber, it was injected into the inlet of the GC-MS QTOF system for five minutes at 250 °C. Prior to breath sampling every day, the SPME fiber was preconditioned at 250 °C for 10 min. The chromatographic protocol for VOC analysis in both methods involved holding the oven temperature at 40 °C for 2 min, followed by a ramp of 8 °C/min to 120 °C, 8 °C/min until 180 °C, 15 °C/min until 200 °C, and a final 8 °C/min ramp to 260 °C. Ultra-high-purity helium was used as the mobile phase with a flow rate of 1.2 mL/min. The mass analyzer was configured for full scanning, with an m/z range from 26 to 400. The ion source was maintained at 250 °C with at least 4 µA emission current. HDPE pellets were also analyzed to monitor instrumental drift throughout experiments, using the same SPME GC-MS method employed for cryothermally transferred breath samples. These external reference standards emit a consistent VOC profile (mostly consisting of saturated and unsaturated hydrocarbon chains) and were used to ensure a sensitive and reproducible analysis. When possible, pure standards were analyzed to confirm preliminary VOC identification, which was initially performed using the NIST17 mass spectral library and an in-house/column-specific nonpolar retention index (NPRI) curve (Schulz et al., 2024). NPRI values were estimated in this study using the retention time of a particular compound and the line of best fit of the previously published calibration curve. Preliminary identification was performed when the estimated NPRI value differed by no more than 100 units from those reported in the NIST17 library.

### Data processing and analysis

**Fig. 1:**
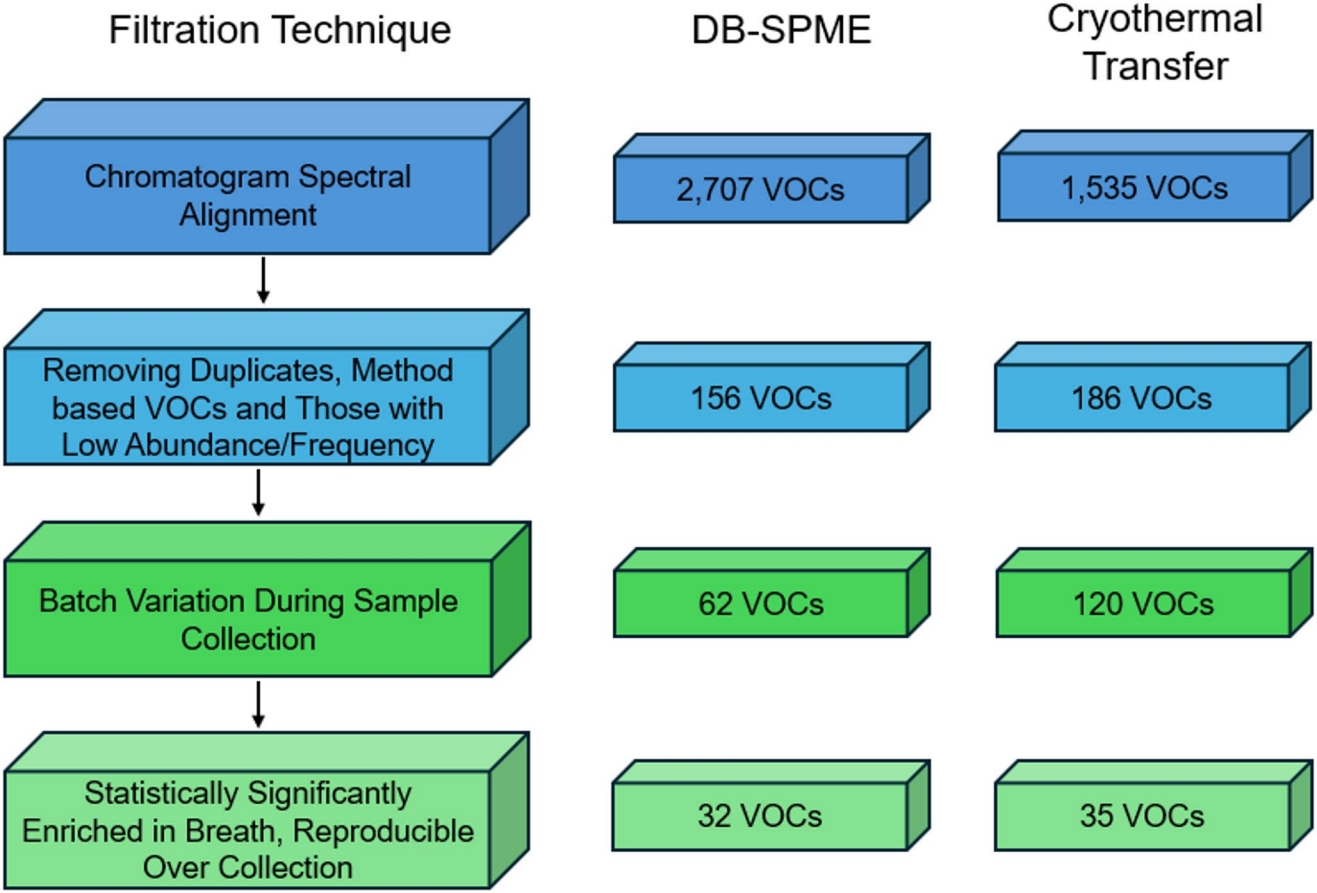
A diagram outlining the VOC filtration steps implemented on both data sets collected using the DB-SPME and cryothermal transfer methods

A flowchart detailing data processing and filtration for both methods is illustrated in Fig. 1. Although data filtration methods mirrored each other for the most part, the data needed to be processed slightly differently. Since DB-SPME is a relatively less sensitive method, breath sample chromatograms were spectrally aligned using an in-house algorithm with VOC signals manually verified using Agilent MassHunter Navigator software through extracted ion chromatograms (EICs) of the base m/z peak. On the other hand, cryothermally transferred samples were spectrally aligned into three batches, corresponding to the time of analysis, using Mass Hunter Quantitative Profinder. A peak height threshold of ≥ 500 was implemented, and compounds required having ≥ 5 peaks with a relative height of ≥ 0.1% of the largest peak. A retention time tolerance of 0.0% ± 0.40 min was used with a minimum dot product of 0.40 between mass spectral vectors and a molecular feature extraction score of ≥ 70. These values allow for VOC matches between samples at a moderate spectral similarity, while preserving compounds that may be lower in signal. Once the three batches were generated, each went through the removal of duplicate rows and method artifacts, allowing for the batches to be stitched into one cryothermal transfer matrix. For data in both methods, the team removed duplicate features along with other VOCs with low abundance (< 10% in all samples including background) or originating from the SPME fiber. Next, the healthy breath data was background subtracted according to the day of analysis, and VOCs that were not present in at least 10% of breath samples with greater signal than the background were removed. Compounds that displayed statistically significant differences across sample batches were removed from analysis, as they likely do not originate from human exhaled breath and would not be useful in analysis.

Finally, VOCs that were not statistically significantly enriched in breath samples relative to the background were removed from the data matrix, which led to the identification of the core set of VOCs in both data sets. It should be noted that cross-sectional data was used for data screening and filtration since it contained a higher number of samples from different individuals. To normalize the VOC data, half of the minimum value (within each method) was added to each of the signals, and the data was winsorized (using a 95% confidence interval) and log_2_ transformed. It should be noted that VOC signals were not log_2_ transformed for reproducibility assessments (through calculation of relative standard deviation (RSD) values). The Mann-Whitney U-Test was utilized to assess VOC differences (including measures of reproducibility) across categorical groups, with *p*-values adjusted via the Bonferroni method to account for false discovery (Bland & Altman, 1995). Linear regression analyses were utilized to assess VOC trends with quantitative variables through Pearson correlation (Microsoft Excel; Version 2506). The Tukey-Kramer multiple comparisons test was used to identify VOC differences between the longitudinal volunteers via GraphPad Prism 10 (Version 10.4.2). Unsupervised multivariate approaches in the form of principal component analysis (PCA) were employed to observe natural patterns within the data and how they correlate with different traits of the study. Hierarchical heatmaps of the healthy breath data were produced in Matlab (Version R2024b Update 4) using a Euclidean distance metric and average linkage to generate the dendrogram.

## Results and discussion

### Breath collection and study demographics

158 volunteers provided cross-sectional breath samples using both methods, and ten of these volunteers donated a total of ten longitudinal samples each over the course of six months (*n* = 158 for cross-sectional study, *n* = 99 for longitudinal study). It can be noted that instrumental analysis of one longitudinal sample was halted due to a GC-MS data acquisition malfunction. Within the cross-sectional cohort, 81 (51%) volunteers identified as female, with the remaining 77 (49%) identifying as male. 44% of this cohort was comprised of White volunteers, 31% were Asian, 9.5% were Black, 9.5% were Hispanic, 1.3% were American Indian/Alaska Native, and 4.4% identified as being other (or declined to answer). The median age range was equal to 27 years, and CMP/CBC data was used to determine the relative health of each volunteer. None of the volunteers were disqualified since there were no significant findings within the CMP/CBC data. Within the longitudinal cohort, eight (80%) volunteers identified as male, and the median age was equal to 27 years. 50% of these volunteers identified as White, 20% as Asian, 20% as Hispanic, and 10% as Black. Supplementary Table S.2 provides an overview of demographic and confounding variable data for both data sets. Due to the study setting being on a college campus, the study cohort represented the typical university population with respect to sex and age distribution. Future studies can perform more targeted approaches for confounders to expand insights on how they impact VOC expression.

### VOC identification in healthy breath

Many breath-based studies report many molecular features (> 1,000) and the current study sought to produce a more reliable data set through applying an array of filters to reduce data dimensionality with parallel methods. The overarching goal of the current study was to employ two different breath sampling methods (DB-SPME and cryothermal transfer) to cross-reference results and thereby increase the fidelity of exhaled breath VOC analysis. Prior to employing any data filters, a total of 2,707 features were identified in the spectrally aligned data matrix for DB-SPME. To reduce the dimensionality of the data, SPME fiber artifacts and duplicate features were removed, which left a total of 156 features. VOCs that were not detected in at least 10% of breath samples with greater intensity than the background were removed, along with compounds significantly varying across the course of collection, resulting in a matrix containing 62 VOCs. Although this may be a lower threshold than what is implemented by most studies, this approach ensured a high degree of coverage regarding breath VOCs. A final set of 32 VOCs were identified to be significantly enriched in DB-SPME breath compared to background (Supplementary Table S.3).

For cryothermal transfer data, three unique alignments were performed according to batch (date of collection), which yielded > 1,000 unique features in each set. The three batches underwent duplicate feature and method artifact removal, and the three batches were manually stitched together using 186 VOCs. This was further reduced to 120 VOCs after focusing on features that were detected in at least 10% of samples and reproducible over collection. Of these VOCs, 35 were significantly enriched in breath (Supplementary Table S.4). Similar data screening procedures have been implemented in research to successfully qualify and identify healthy on-breath VOCs (Arulvasan et al., 2024). It is important to note that for a handful of features in this matrix, specific names were not attained through our identification process, and therefore they are listed based on their primary functional group or as unknowns. Although our previous study determined cryothermal transfer to be more sensitive than DB-SPME, a similar number of features were observed between the methods post-filtration. This may be because the Bonferroni method results in a stricter *p*-value threshold as the number of observations increases. In the future, less aggressive or even non-adjusted *p*-values can be considered and assessed in VOC studies.

Although a similar number of features were detected between the two methods, the classes of VOCs observed with respect to molecular weight were markedly different. For instance, 67% of VOCs detected in DB-SPME were between an NPRI range of 550–1041. The cryothermal transfer method on the other hand only detected 31% of its VOCs within this range, with 67% of its VOCs being identified within an NPRI range of 898–1646, demonstrating the increased sensitivity of the method towards heavier weight VOCs. The benefit of using parallel methods is highlighted in this case, as one method may miss out on VOCs that the other can detect. There are two plausible explanations for the reduced sensitivity of the cryotransfer method to certain VOCs with low NPRI. First, smaller analytes may be lost during the vacuum-assisted cryotransfer process, as their volatility and low molecular weight make them more susceptible to evaporation or incomplete capture. Second, the extraction temperature of 60 °C used in the cryothermal transfer method may favor the adsorption of larger, more hydrophobic VOCs onto the SPME fiber, thereby competitively inhibiting the capture of smaller compounds (Schulz et al., 2023). When assessing the functional group frequencies detected by both methods, there was a high degree of structural similarity in VOCs detected (Supplementary Figure S.2). Some of the most abundant functional groups in both data sets include nonaromatic cyclic VOCs as well as terpenes/terpenoids.

### Intra- and inter-method VOC correlations

**Fig. 2:**
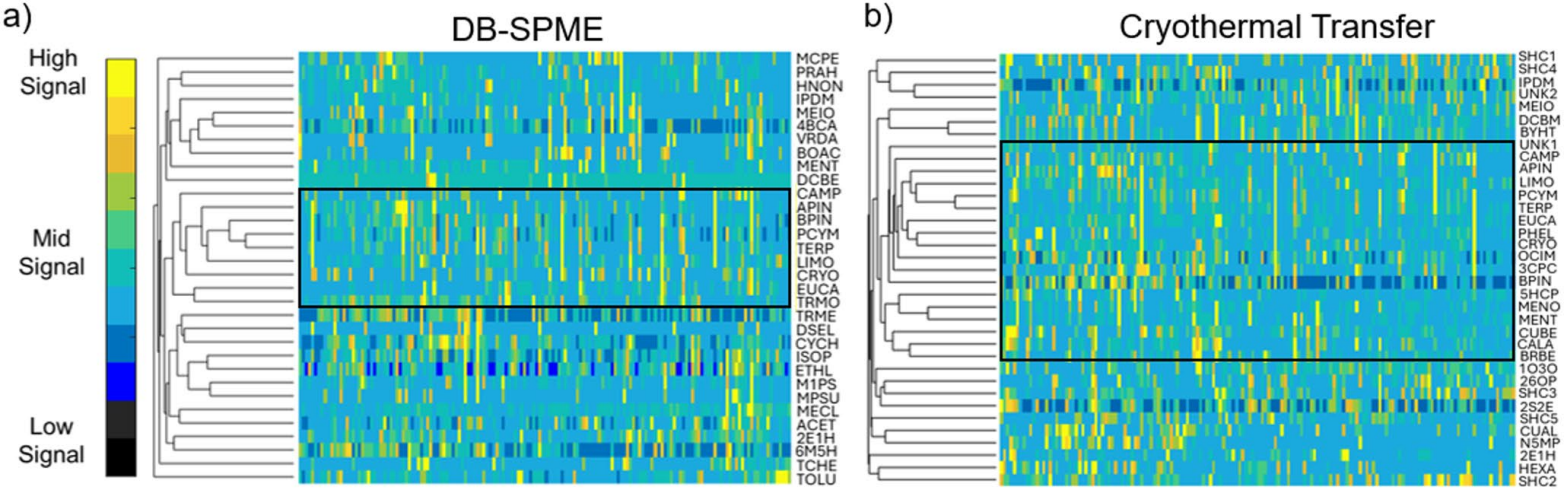
Hierarchical heatmaps of cross-sectional data from both **a** DB-SPME and **b** cryothermal transfer methods showing the clustering of terpene/terpenoid VOCs (outlined in black) based on their levels of expression in collected samples

**Fig. 3:**
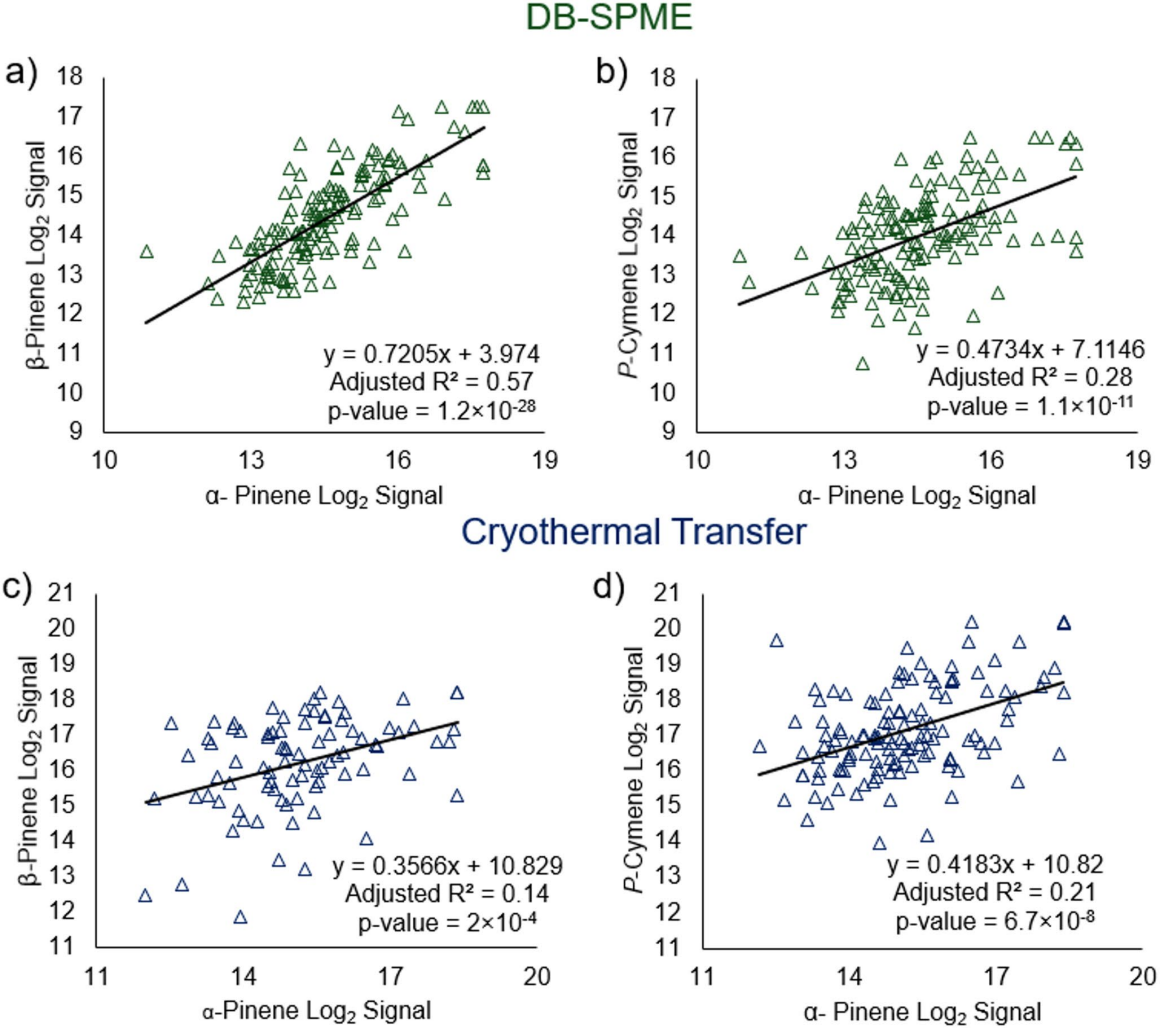
Scatter plots showing the correlation of VOCs between one another within a given data set. Statistically significant correlations are shown for both α-pinene/β-pinene and α-pinene/p-cymene using **a**, **b** DB-SPME and **c**, **d** cryothermal transfer methods

After the core VOCs were determined in both methods, hierarchical heatmaps were constructed using the cross-sectional samples. The dendrograms on the left side of the heatmaps illustrate how different VOCs cluster based on similarities in signal within the sample data. Interestingly, structurally similar terpenes (including camphene, α-pinene, β-pinene, p-cymene, γ-terpinene, limonene, β-caryophyllene, and eucalyptol) clustered together and showed analogous trends within both data matrices (outlined with black in Fig. 2). To further investigate similarities in terpene expression, correlation matrices were produced which revealed moderate statistically significant correlations among terpenes (Supplementary Figures S.3 and S.4). For DB-SPME, terpene correlations have been highlighted including but not limited to α-pinene/β-pinene (slope = 0.72 [95% CI (0.62–0.82)], y-intercept = 3.97 [95% CI (2.48–5.46)], Adjusted R^2^ = 0.57, *p* = 1.2 × 10^−28^) in Fig. 3(a) and α-pinene/p-cymene (slope = 0.47 [95% CI (0.35–0.60)], y-intercept = 7.11 [95% CI (5.26–8.97)], Adjusted R^2^ = 0.28, *p =* 1.1 × 10^−11^) in Fig. 3(b). Scatter plots in Fig. 3 are only with samples that had detectable signals, while correlation matrices were generated using all samples. The same VOC correlations were illustrated for the cryothermal transfer dataset, with α-pinene/β-pinene (slope = 0.36 [95% CI (0.17–0.54)], y-intercept = 10.83 [95% CI (8.05–13.61)], Adjusted R^2^ = 0.14, *p =* 2.0 × 10^−4^) in Fig. 3(c) and α-pinene/p-cymene (slope = 0.42 [95% CI (0.27–0.56)], y-intercept = 10.82 [95% CI (8.63–13.01)], Adjusted R^2^ = 0.21, *p =* 6.7 × 10^−8^) in Fig. 3(d). Cryothermal transfer generally displays less significant correlations relative to DB-SPME, which is more than likely due to the inherently greater method variability. Our previous study showed that DB-SPME facilitated a more reproducible analysis compared to the cryothermal transfer method (Schulz et al., 2023), and this may be the reasoning for these results which were observed in this study.

**Fig. 4:**
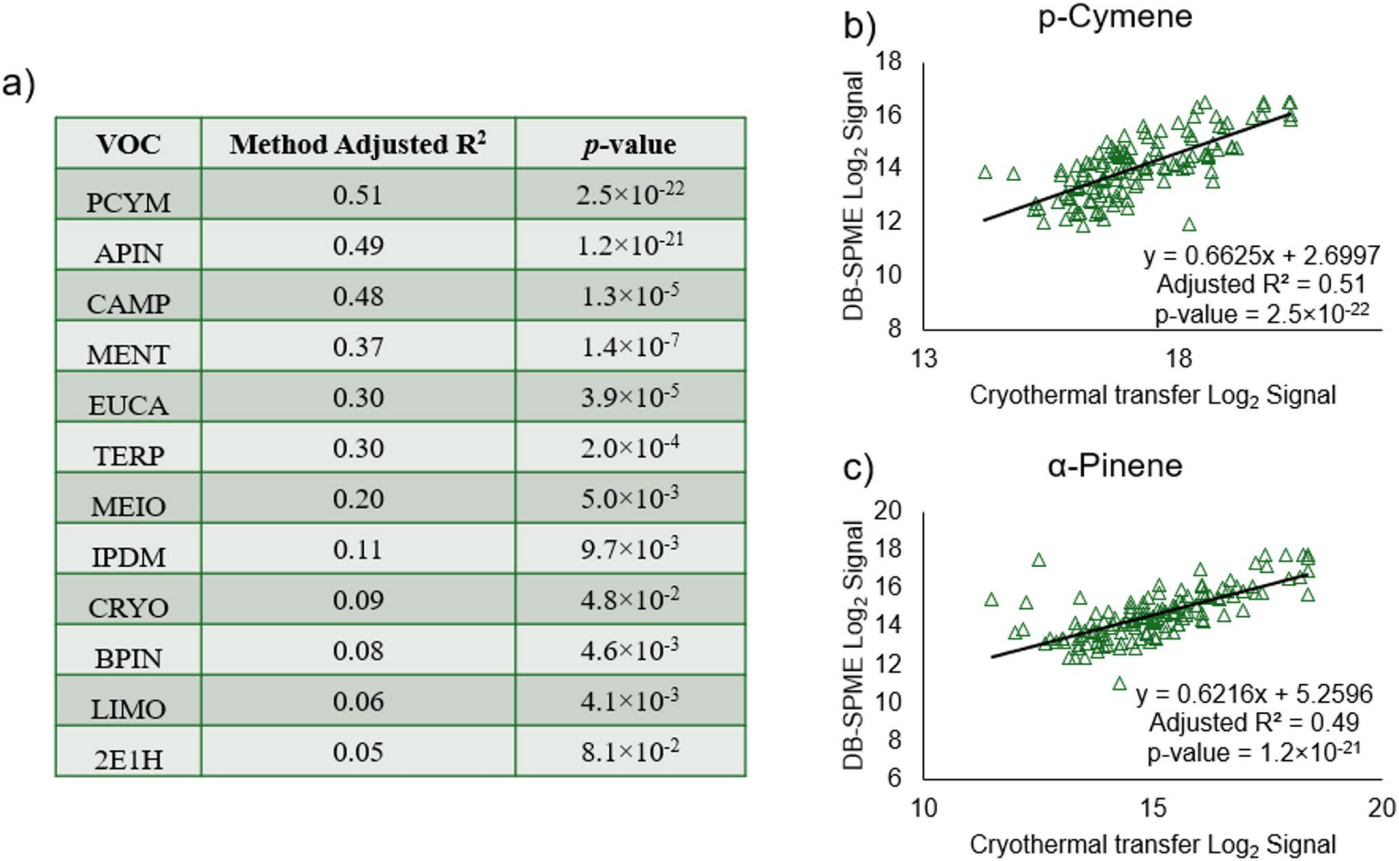
Common VOCs between both methods were screened, resulting in **a** 12 compounds being detected in both DB-SPME and cryothermal transfer, with 11 showing statistically significant correlations. Scatter plots correlating specific VOCs among the two methods are shown for **b** p-cymene and **c** α-pinene

Given the structural similarity of these compounds (volatile terpenes and terpenoids) and their observed clustering, it is likely that they have a similar source. Previous research has postulated that volatile terpenes including limonene are exogenously introduced into the body but can still be a valuable probe for the evaluation of liver disease based on endogenous interactions with cytochrome P450 enzymes (Ferrandino et al., 2023). Volatile terpenes/terpenoids can have exogenous sources including but not limited to different foods (plants/vegetables), beverages, as well as other consumer products (essential oils, fragrances, scents, etc.). In addition to terpenes and terpenoids, other compounds with likely exogenous origins include dimethyl selenide—a volatile metabolite excreted as a byproduct of dietary selenium (micronutrient commonly found in many foods) (Van den Velde et al., 2008). To cross-validate findings and improve the robustness of presented results, 12 features between the two methods were identified via retention time, NPRI, and base m/z (including α-pinene, camphene, β-pinene, p-cymene, 2-ethyl-1-hexanol, limonene, eucalyptol γ-terpinene, menthol, β-caryophyllene, methyl ionone, and (7a-isopropenyl-4,5-dimethyloctahydroinden-4-yl)methanol). The coefficient of determination for these VOCs between the two methods are displayed in Fig. 4(a). Two of the stronger correlations were shown to be with p-cymene (slope = 0.66 [95% CI (0.55–0.77)], y-intercept = 2.70 [95% CI (0.78–4.61)], Adjusted R^2^ = 0.51, *p =* 2.5 × 10^−22^) and α-pinene (slope = 0.62 [95% CI (0.51–0.73)], y-intercept = 5.26 [95% CI (3.64–6.88)], Adjusted R^2^ = 0.49, *p =* 1.2 × 10^−21^) which are illustrated in Fig. 4(b) and (c). Combined with the hierarchical clustering of the terpene/-oids in each method, displaying correlations of VOCs between both methods increases the confidence of observing these analytes and their quantitative trends in biological data sets. In other words, showing consistent VOC correlations across both methods help strengthen confidence in the quantitative results presented through cross-validation.

### Benchmarking VOC variability

**Fig. 5:**
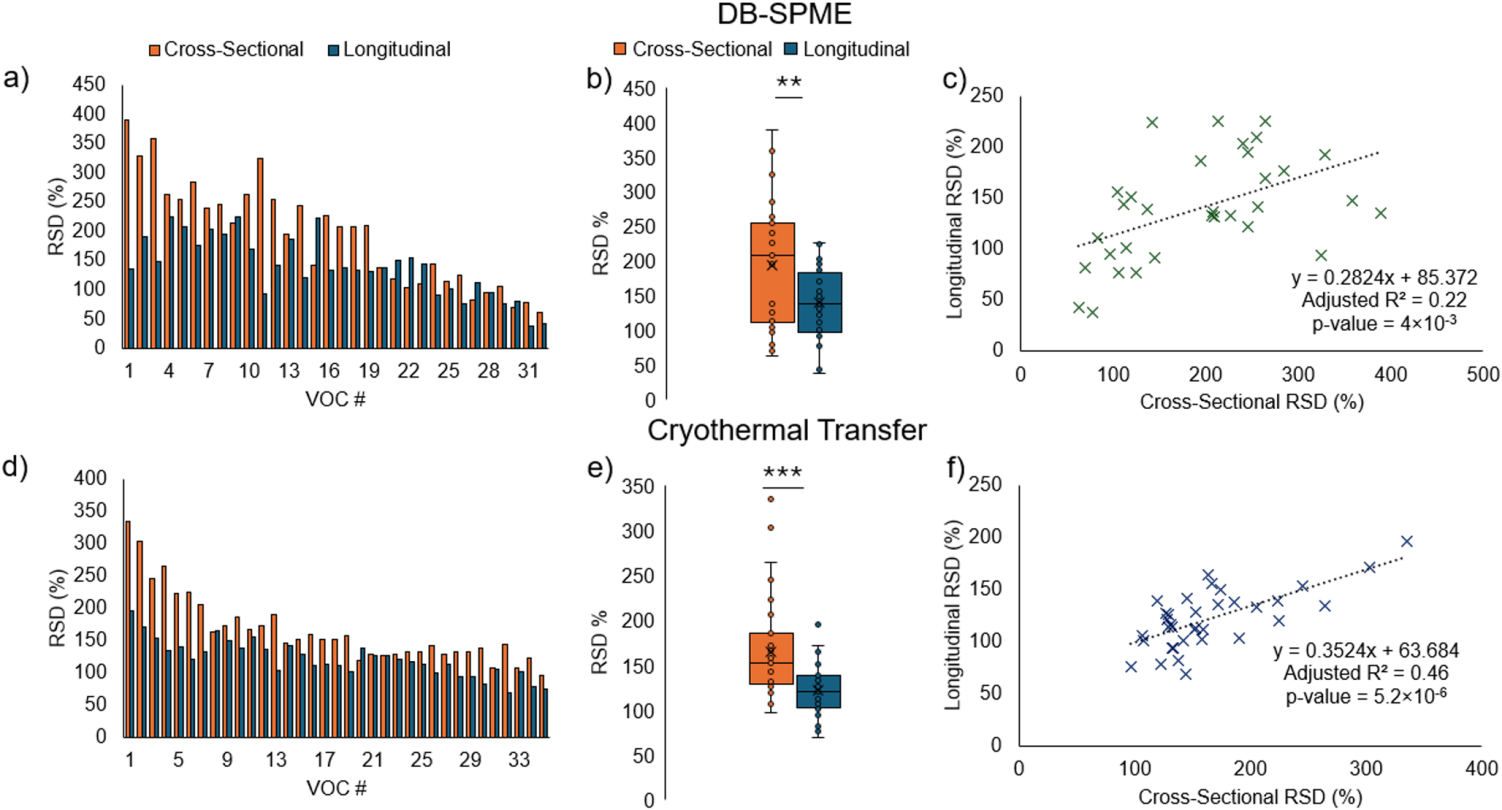
Reproducibility assessments performed on cross-sectional and longitudinal samples. **a**, **d** Bar plots, **b**, **e** box/whisker plots, and **c**, **f** scatter plots showing degrees of correlations are provided for both DB-SPME and cryothermal transfer methods (*p-value < 0.05, **p-value < 0.01, ***p-value < 0.001)

It is important to take into account inherent method-based variability, especially when considering the VOC signal variation that may be observed in this study. As published previously, Generally DB-SPME had an RSD range of 12–44%, whereas cryothermal transfer had an RSD range of 16–134%. To assess variability in the identified VOCs in this study, RSDs were calculated in the cross-sectional and longitudinal samples for both data sets. It should be noted that for VOCs in the longitudinal data, the RSD was calculated for each volunteer, and an average was calculated across all 10 volunteers. For both sample cohorts, bar plots, box/whisker plots and scatter plots are shown to compare and correlate cross-sectional and longitudinal RSD measurements. Across both data sets, it can be observed that there is a significantly increased VOC reproducibility in longitudinal samples compared to cross-sectional (Seen in Fig. 5(a-b) for DB-SPME and Fig. 5(d-e) for cryothermal transfer). For DB-SPME samples (*p*-value = 0.005), the cross-sectional RSD mean was equal to 194.15 [95% CI (161.68-226.63)] while the longitudinal RSD mean was 140.20 [95% CI (121.75-158.65)]. Cryothermally transferred samples on the other hand (*p*-value = 0.0001), had cross-sectional RSD mean equal to 165.56 [95% CI (146.76-184.36)] and longitudinal RSD mean equal to 122.03 [95% CI (112.39-131.68)]. Correlation analyses on the other hand revealed that there was a significant correlation, where VOCs that were more variable in the cross-sectional data were also generally more variable longitudinally (Fig. 5(c) for DB-SPME (slope = 0.28 [95% CI (0.10–0.47)], y-intercept = 85.37 [95% CI (46.13-124.61)], Adjusted R^2^ = 0.22, *p =* 4.0 × 10^−3^) and Fig. 5(f) for cryothermal transfer (slope = 0.35 [95% CI (0.22–0.48)], y-intercept = 63.68 [95% CI (40.70-86.67)], Adjusted R^2^ = 0.46, *p =* 5.2 × 10^−6^). Although the RSD values for some VOCs may be high, the mirrored characteristics between the methods which are cross-referenced (increased longitudinal reproducibility and consistent VOC correlations in RSD between cross-sectional and longitudinal analyses) build confidence in the quantitative VOC analyses. Further enhancements to sampling methods can certainly be made in the future to further reduce the variation that may be contributed by these methods. For example, fractionating exhaled breath into Tedlar bags and transferring VOCs in a standardized manner to a sorbent tube may significantly reduce variability introduced by the analytical method (Woollam et al., 2025b).

### Biological characteristics of sample cohort

**Fig. 6:**
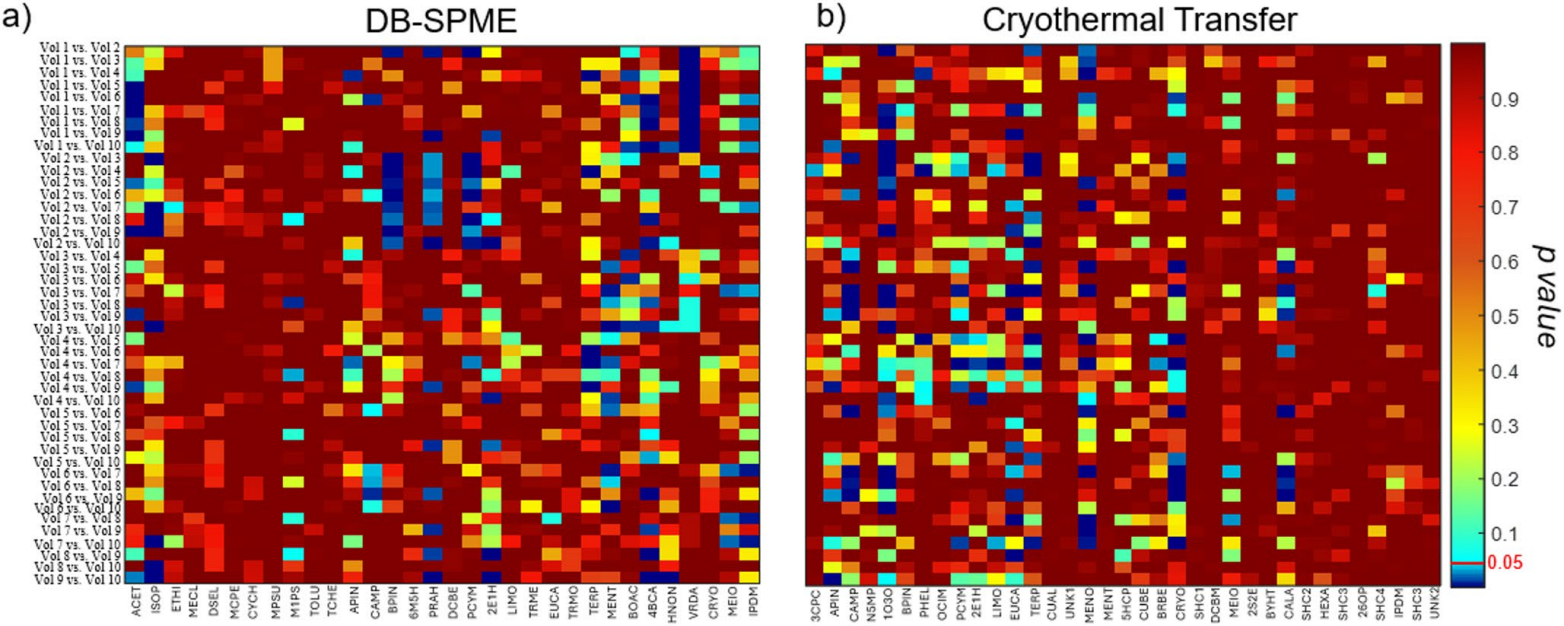
Heatmap of p-values were generated to show any significant differences between the longitudinal volunteers in both DB-SPME and cryothermal transfer data sets. Teal and blue colored boxes represent comparisons that are statistically significant (*p*-value < 0.05)

To profile longitudinal differences that may be reflective of biological variation, the Tukey-Kramer multiple comparisons test was performed, with both datasets undergoing 45 comparisons for each VOC to ensure each longitudinal volunteer was compared to one another (heatmap of *p*-values in Fig. 6). Within the DB-SPME matrix, 18 out of the 32 VOCs demonstrated at least one statistically significant difference between volunteers. Propanoic acid anhydride was most prominent, having 16 differences within the cohort (Fig. 6(a)). The remaining VOCs include but are not limited to acetone, isoprene, p-cymene, α-pinene, camphene, eucalyptol, and menthol. In the cryothermal transfer dataset, 15 out of 35 VOCs showed at least one difference, with camphene and eucalyptol having 11 differences within the cohort (Fig. 6(b)). Other compounds that showed differences among the comparisons included but were not limited to α-pinene, camphene, limonene, and menthone. Overall, the authors suggest that VOCs that demonstrate differences between volunteers will be the analytes that are most relevant in a biological context.

To further investigate biological variation, analysis of confounding variables was performed in cross-sectional data sets, and little to no correlations were observed except for isoprene and ethanethiol for biological sex in DB-SPME data. The lack of correlation to confounding variables can be explained through the limited diversity of study characteristics. In other words, when a confounding variable lacks sufficient variation, identifying correlations with VOCs becomes difficult or in some cases unachievable. For example, over 70% of the volunteers were 30 years of age or younger, making it difficult to elucidate VOC changes. This agrees with a recent study that demonstrated that the most significant shifts in breath profiles occur at around age 32 (Sasiene et al., 2024). Consumption of plants and foods is also suspected to contribute to the variation in VOC expression (primarily terpenes/terpenoids), although breath-based data in this study showed little to no correlation with specific diets or dietary restrictions. This may be because volatile terpenes/terpenoids could have multiple exogenous sources beyond that of food, and this is difficult to monitor. Circadian rhythms have also been classified as contributors to observed VOC variation, and although this study did not collect dedicated information for this purpose, future studies could aim to correlate exhaled breath VOCs to bloodborne markers including cortisol and melatonin.

Nonetheless, volunteers who identified themselves as male had statistically elevated levels of isoprene relative to women (*p* = 0.0006, fold change = 1.03), and this is shown in Supplementary Figure S.5(a). The mean log_2_-normalized isoprene signal for women was equal to 17.31 [95% CI (17.13–17.50)], while for males it was equal to 17.79 [95% CI (17.59–17.99)]. Even though isoprene was significantly elevated in males, PCA of the 32 core VOCs (DB-SPME) was unable to distinguish males from females (Figure S.5(b). The observed increases in isoprene levels among males may be attributed to elevated muscular lipolytic cholesterol metabolism. Previous literature has identified enriched isoprene signals in the breath of male volunteers (Lechner et al., 2006), and in general men have statistically significantly more skeletal muscle mass relative to women (Janssen et al., 2000). A recent study investigated the metabolic origin of isoprene using a multi-omic approach (Sukul et al., 2023) and showed that endogenous production is correlated with isopentenyl-diphosphate delta isomerase 2 (IDI2) protein expression. This challenges the notion that isoprene (and perhaps other terpene/terpenoid VOCs) is generated in the liver, as IDI2 is only expressed in skeletal-myocellular peroxisomes through muscular lipolytic cholesterol metabolism. In addition to isoprene, ethanethiol was also identified to be significantly upregulated in men compared to women (*p* = 0.0004, fold change = 1.22). The mean log_2_-normalized ethanethiol signal for women was equal to 10.22 [95% CI (9.15–11.29)] and for men it was equal to 12.47 [95% CI (11.85–13.08)]. It should be noted that further studies are required to provide a robust biological rationale for this result.

## Conclusion

In summary, the current research makes strides to develop a more fundamental understanding on the chemical composition and variability of VOC profiles in breath samples collected from a relatively healthy population. Different core sets of breath VOCs were identified through use of two unique breath sampling methods, which ultimately resulted in high-quality quantitative analyses (VOC correlations, assessment of reproducibility, etc.). The presented findings demonstrate the benefit of performing parallel sampling methods especially in an untargeted VOC study, to cross-reference results and decrease the chance of false discovery. To translate the potential of VOCs into clinical practice, because there is no gold standard technique, the authors recommend running samples using at least two breath sampling methods. Additionally, mutli-center studies or community-initiated breath-based databases may be able to overcome current challenges regarding validation of results, as the field furthers its development to a gold-standard sampling technique. Until a universally accepted gold standard for offline breath sampling is established, this study highlights the value of using multiple sampling strategies to cross-validate findings, thereby strengthening confidence in VOC results.

## Supplementary Information

Below is the link to the electronic supplementary material.

Supplementary material 1 (DOCX 594.8 kb)

## Data Availability

The authors provide no restriction on the availability of methods, protocols, instrumentation and data utilized in the following article. All data will be available from the corresponding author by request.
