# Supplementary material for: Parallel Offline Breath Sampling for Cross-Validated Analysis of Volatile Organic Compound Metabolites
Source: Metabolomics. 2025 Sep 17;21(5):138. doi: 10.1007/s11306-025-02340-1 (PMC12443928; doi:10.1007/s11306-025-02340-1)
Supplement: Supplementary file 1 — Supplementary material 1 (DOCX 594.8 kb) [file 11306_2025_2340_MOESM1_ESM.docx]

**Title:** Parallel Offline Breath Sampling for Cross-Validated Analysis of Volatile Organic Compound Metabolites

**Authors:** Eray Schulz^1,2^, Mariana Maciel^2^, Zhige Wang^3^, Shivaum Heranjal^2,4^, Xiaowen Liu^5^, Sha Cao^6^, Ryan F. Relich^7^, Mark Woollam^1,2*^, and Mangilal Agarwal^1,2,8*^

**Institutions:**

1. Chemistry & Chemical Biology, Indiana University Indianapolis, IN, United States.
2. Integrated Nanosystems Development Institute, Indiana University Indianapolis, IN, United States.
3. Department of Computer Science, Tulane University School of Engineering, New Orleans, LA, United States.
4. Electrical & Computer Engineering, Purdue University Indianapolis, IN, United States.
5. Deming Department of Medicine, Tulane University School of Medicine, New Orleans, LA, United States.
6. Biomedical Engineering, Oregon Health & Science University School of Medicine, Portland, OR, United States.
7. Pathology & Laboratory Medicine, Indiana University School of Medicine, Indianapolis, IN, United States.
8. Biomedical Engineering & Informatics, Luddy School of Informatics, Indiana University Indianapolis, IN, United States.

Table S.1 Manufacturer and part number information for the materials used in the presented study.

| **Material/Item** | **Manufacturer** | **Manufacturer Part Number** |
| --- | --- | --- |
| Viral Filter | ViroMax | 156200 |
| NM3 Capnograph | Phillips | 168-00202 |
| Glass Pasteur Pipettes | Fisher | 13-678-20C |
| Tygon Tubing | Saint-Gobain | 00067 |
| Tedlar Bags | Restek | 22051 |
| Mass Flow Controller | Alicat | 97069 |
| Hypodermic Needles | Jorvet | JOR959 |
| Headspace Vials/Caps | Restek | 5188-6537/319557 |
| Gas Chromatograph/Mass Spectrometer | Agilent | G3330A/G3850-89201 |
| PAL Autosampler | CTC Analytics | G7368-64100 |
| SPME Fibers | Supelco | 57348-U |
| HDPE Pellets | The Fundamental Rockhound | N/A |


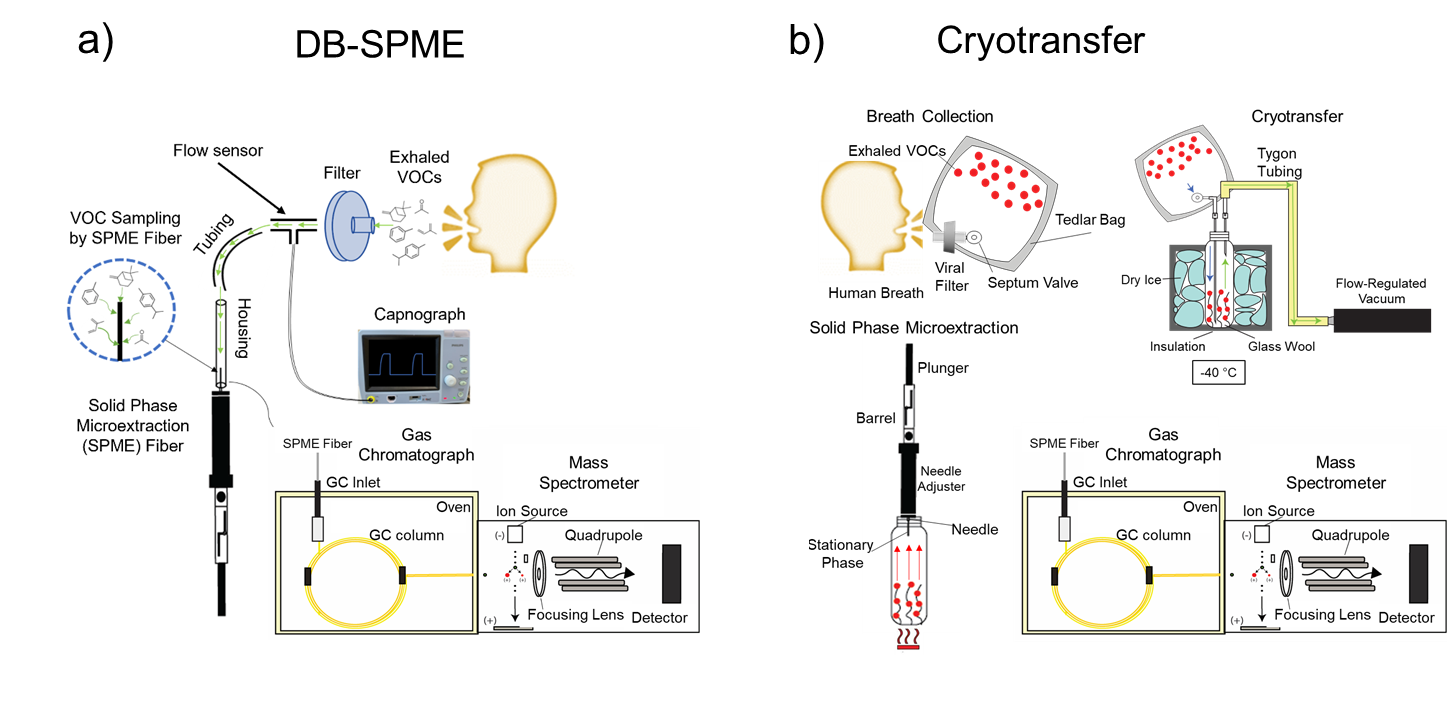


Figure S.1. Two breath sampling methods were utilized to capture and analyze exhaled breath VOCs. A fast and relatively easy sampling method known as (a) DB-SPME was used, followed by (b) a Tedlar bag-based method known as cryothermal transfer (cryotransfer), where exhaled breath collected in a bag is transferred via vacuum into a headspace vial.

Table S.2. Demographic and confounding variable data for the cross-sectional and longitudinal sample cohorts.

| **Characteristics** | **Cross-sectional (*N*^b^ *=* 158)** | **Longitudinal (*N =* 10, n=99)** |
| --- | --- | --- |
| Age, years, median ± SD^c^ | 27.0 ± 11.6 | 27.0 ± 3.0 |
| Body Mass Index, kg/m^2^, medians (IQR^a^) | 25.1 (23.0-29.0) | 23.7 ± (19.7-25.2) |
| Sex, Female, (%) | 51.0 | 20.0 |
| Sex, Male, (%) | 49.0 | 80.0 |
| Smoking history, (%) | 16.0 | 10.0 |
| Special/Restricted Diet, (%) | 19.0 | 10.0 |
| Sleep, hours, means ± SD | 7.0 ± 1.0 | 6.8 ± 0.8 |

^a^IQR: interquartile range

^b^N: number

^c^SD: standard deviation.

Table S.3. Summary statistics and other identifiers for the core 32 VOCs identified using the DB-SPME sampling method. The compound abbreviations, m/z, NPRI, CAS# (if verified by standards), whether they were verified by standards, Metabolomics Standards Initiative (MSI) identification level, and frequency of detection (%) in the cross-sectional cohort are provided.

| **VOC** | **Abbreviation** | **Base m/z^a^** | **Secondary m/z** | **NPRI^b^** | **CAS#^c^** | **Verified by Standards** | **MSI^d^ Classification** | **Frequency (%)** |
| --- | --- | --- | --- | --- | --- | --- | --- | --- |
| Acetone | ACET | 43 | 58 | 550.1 | 67-64-1 | Yes | 1^st^ Level | 100 |
| Isoprene | ISOP | 67 | 68 | 552.9 | 78-79-5 | Yes | 1^st^ Level | 100 |
| Ethanethiol | ETHL | 62 | 46 | 555.4 | 75-08-1 | Yes | 1^st^ Level | 68 |
| Methylene Chloride | MECL | 49 | 84 | 558.4 | 75-09-2 | Yes | 1^st^ Level | 62 |
| Dimethyl Selenide | DSEL | 110 | 95 | 572.5 | 593-79-3 | Yes | 1^st^ Level | 7 |
| Methyl cyclopentane | MCPE | 56 | 82 | 592.6 | 96-37-7 | Yes | 1^st^ Level | 21 |
| Cyclohexane | CYCH | 56 | 84 | 613.4 | 71-43-2 | Yes | 1^st^ Level | 59 |
| Methyl Propyl Sulfide | MPSU | 61 | 90 | 652.2 | 3877-15-4 | Yes | 1^st^ Level | 12 |
| 1-(Methylthio)-1-propene | M1PS | 88 | 73 | 673.0 | X | No | 2^nd^ Level | 20 |
| Toluene | TOLU | 91 | 77 | 706.6 | 108-88-3 | Yes | 1^st^ Level | 93 |
| Tetrachloroethylene | TCHE | 166 | 128 | 756.4 | 127-18-4 | Yes | 1^st^ Level | 19 |
| α-Pinene | APIN | 93 | 91 | 918.5 | 80-56-8 | Yes | 1^st^ Level | 100 |
| Camphene | CAMP | 93 | 91 | 938.1 | 79-92-5 | Yes | 1^st^ Level | 15 |
| β-Pinene | BPIN | 93 | 91 | 975.1 | 18172-67-3 | Yes | 1^st^ Level | 94 |
| 6-Methyl-5-hepten-2-one | 6M5H | 93 | 108 | 986.6 | 110-93-0 | Yes | 1^st^ Level | 46 |
| Propanoic anhydride | PRAH | 57 | 86 | 991.6 | X | No | 2^nd^ Level | 28 |
| 1,3-Dichlorobenzene | DCBE | 146 | 110 | 1020.0 | 106-46-7 | Yes | 1^st^ Level | 9 |
| p-Cymene | PCYM | 119 | 67 | 1035.9 | 99-87-6 | Yes | 1^st^ Level | 90 |
| 2-Ethyl-1-hexanol | 2E1H | 57 | 41 | 1039.4 | 104-76-7 | Yes | 1^st^ Level | 57 |
| Limonene | LIMO | 67 | 93 | 1041.9 | 5989-27-5 | Yes | 1^st^ Level | 95 |
| 3,3,4-Trimethylheptane | TRME | 57 | 67 | 1043.7 | X | No | 2^nd^ Level | 40 |
| Eucalyptol | EUCA | 93 | 81 | 1045.0 | 470-82-6 | Yes | 1^st^ Level | 42 |
| 2,6,6-Trimethyloctane | TRMO | 71 | 43 | 1048.1 | X | No | 2^nd^ Level | 25 |
| γ-Terpinene | TERP | 93 | 119 | 1077.4 | 99-85-4 | Yes | 1^st^ Level | 27 |
| Menthol | MENT | 81 | 95 | 1194.6 | 2216-51-5 | Yes | 1^st^ Level | 49 |
| Bornyl acetate | BOAC | 95 | 121 | 1305.6 | 5655-61-8 | Yes | 1^st^ Level | 24 |
| 4-tert-Butylcyclohexyl acetate | 4BCA | 67 | 82 | 1310.1 | X | No | 2^nd^ Level | 58 |
| 2,2,4,4,6,8,8-Heptamethylnonane | HNON | 57 | 71 | 1336.7 | 4390-04-9 | Yes | 1^st^ Level | 80 |
| Verdyl acetate | VRDA | 66 | 91 | 1431.3 | 5413-60-5 | Yes | 1^st^ Level | 18 |
| β-Caryophyllene | CRYO | 91 | 105 | 1433.6 | 87-44-5 | Yes | 1^st^ Level | 26 |
| Methyl ionone | MEIO | 135 | 43 | 1483.3 | X | No | 2^nd^ Level | 18 |
| (7a-Isopropenyl-4,5-dimethyloctahydroinden-4-yl) methanol | IPDM | 191 | 119 | 1646.0 | X | No | 2^nd^ Level | 28 |

^a^Base m/z: Base mass-to-charge ratio

^b^NPRI: Non-Polar Retention Index

^c^CAS#: Chemical Abstracts Service Number

^d^MSI Classification: Metabolomics Standards Initiative Level

Table S.4. Summary statistics and other identifiers for the core 35 VOCs identified using the cryothermal transfer sampling method. The compound abbreviations, m/z, NPRI, CAS# (if verified by standards), whether they were verified by standards, Metabolomics Standards Initiative (MSI) identification level, and frequency of detection (%) in the cross-sectional cohort are provided. VOCs with functional group names were unable to be reasonably identified using NIST and NPRI calibration curves.

| **VOC** | **Abbreviation** | **Base m/z^a^** | **Secondary m/z** | **NPRI^b^** | **CAS#^c^** | **Verified by Standards** | **MSI^d^ Classification** | **Frequency (%)** |
| --- | --- | --- | --- | --- | --- | --- | --- | --- |
| 3-Chloropropionyl chloride | 3CPC | 93 | 91 | 898.2 | X | No | 2^nd^ Level | 37 |
| α-Pinene | APIN | 93 | 91 | 918.5 | 80-56-8 | Yes | 1^st^ Level | 87 |
| Camphene | CAMP | 93 | 79 | 926.6 | 79-92-5 | Yes | 1^st^ Level | 42 |
| 5-Methyl-5-propylnonane | N5MP | 57 | 71 | 953.8 | X | No | 2^nd^ Level | 23 |
| 1-Octen-3-ol | 1O3O | 57 | 43 | 963.7 | X | No | 2^nd^ Level | 52 |
| β-Pinene | BPIN | 93 | 91 | 975.1 | 18172-67-3 | Yes | 1^st^ Level | 61 |
| Phellandrene | PHEL | 93 | 91 | 997.1 | X | No | 2^nd^ Level | 32 |
| Ocimene | OCIM | 93 | 91 | 1006.3 | X | No | 2^nd^ Level | 87 |
| p-Cymene | PCYM | 119 | 67 | 1035.9 | 99-87-6 | Yes | 1^st^ Level | 88 |
| 2-Ethyl-1-hexanol | 2E1H | 57 | 41 | 1039.4 | 104-76-7 | Yes | 1^st^ Level | 36 |
| Limonene | LIMO | 67 | 93 | 1041.9 | 5989-27-5 | Yes | 1^st^ Level | 88 |
| Eucalyptol | EUCA | 93 | 81 | 1045.0 | 470-82-6 | Yes | 1^st^ Level | 72 |
| γ-Terpinene | TERP | 93 | 119 | 1077.4 | 99-85-4 | Yes | 1^st^ Level | 73 |
| Cumyl alcohol | CUAL | 135 | 105 | 1093.5 | X | No | 2^nd^ Level | 33 |
| Unknown 1 | UNK1 | 57 | 43 | 1097.2 | X | No | 4^th^ Level | 38 |
| Menthone | MENO | 112 | 139 | 1176.0 | X | No | 2^nd^ Level | 78 |
| Menthol | MENT | 81 | 95 | 1194.6 | 2216-51-5 | Yes | 1^st^ Level | 49 |
| 5-Hexyl-3,3-dimethylcyclopentene | 5HCP | 95 | 81 | 1294.9 | 61142-66-3 | No | 2^nd^ Level | 40 |
| Cubebene | CUBE | 105 | 119 | 1391.4 | X | No | 2^nd^ Level | 57 |
| Bourbonene | BRBE | 81 | 123 | 1387.0 | X | No | 2^nd^ Level | 49 |
| β-Caryophyllene | CRYO | 91 | 105 | 1433.6 | 87-44-5 | Yes | 1^st^ Level | 66 |
| Saturated HC 1 | SHC1 | 57 | 71 | 1445.1 | X | No | 3^rd^ Level | 51 |
| 3,4-Dichlorobenzenamine | DCBM | 161 | 203 | 1467.3 | X | No | 2^nd^ Level | 58 |
| Methyl ionone | MEIO | 135 | 43 | 1646.0 | X | No | 2^nd^ Level | 49 |
| 2-ethylhexyl isohexyl sulfite | 2S2E | 57 | 95 | 1485.3 | X | No | 2^nd^ Level | 63 |
| Butylated hydroxytoluene | BYHT | 205 | 220 | 1510.6 | 128-37-0 | Yes | 1^st^ Level | 62 |
| Calamene | CALA | 159 | 131 | 1510.0 | X | No | 2^nd^ Level | 42 |
| Saturated HC 2 | SHC2 | 57 | 71 | 1513.7 | X | No | 3^rd^ Level | 53 |
| Hexadecane | HEXA | 57 | 71 | 1548.9 | X | No | 2^nd^ Level | 32 |
| Saturated HC 3 | SHC3 | 57 | 71 | 1560.0 | X | No | 3^rd^ Level | 37 |
| 2,6-Bis(1,1-dimethylethyl)-4-(1-oxopropyl)phenol | 26OP | 233 | 95 | 1602.7 | X | No | 2^nd^ Level | 44 |
| Saturated HC 4 | SHC4 | 57 | 71 | 1611.3 | X | No | 3^rd^ Level | 35 |
| (7a-isopropenyl-4,5-dimethyloctahydroinden-4-yl) methanol | IPDM | 191 | 119 | 1630.5 | X | No | 2^nd^ Level | 71 |
| Saturated HC 5 | SHC5 | 57 | 190 | 1649.0 | X | No | 3^rd^ Level | 39 |
| Unknown 2 | UNK2 | 57 | 71 | 1652.7 | X | No | 4^th^ Level | 46 |

^a^Base m/z: Base mass-to-charge ratio

^b^NPRI: Non-Polar Retention Index

^c^CAS#: Chemical Abstracts Service Number

^d^MSI Classification: Metabolomics Standards Initiative Level

**
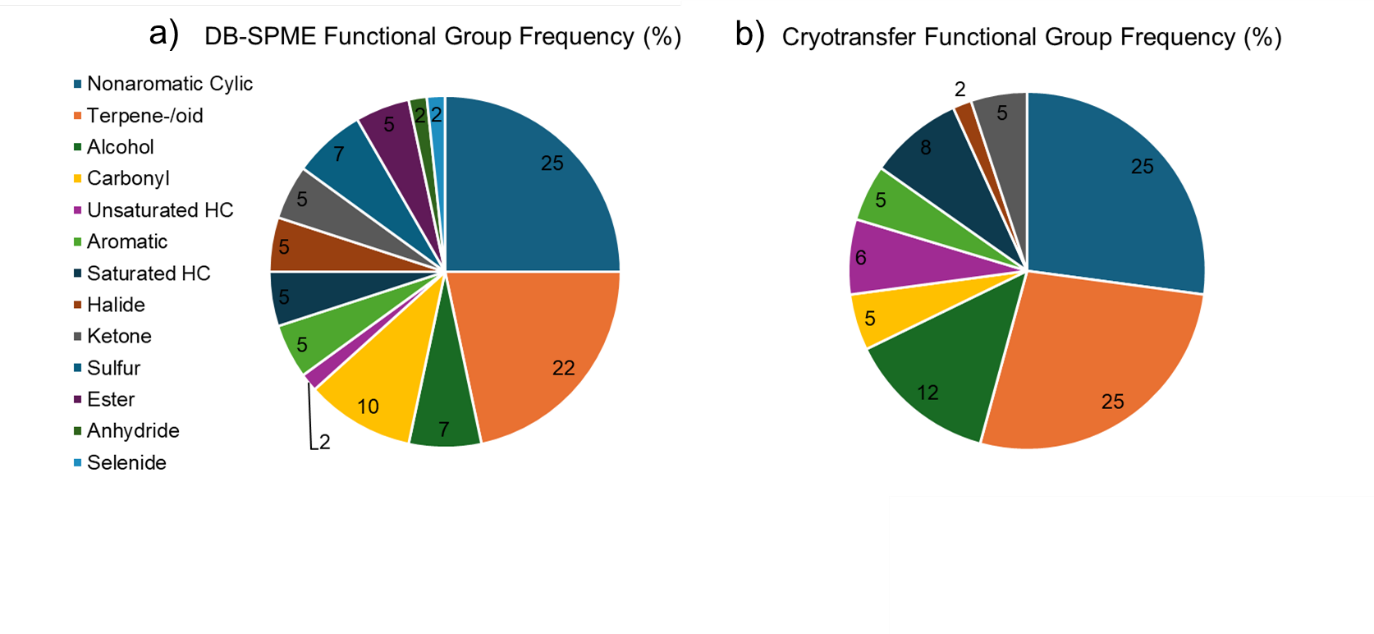
**

Figure S.2. Pie charts of the functional group families for identified VOCs from (a) DB-SPME and (b) cryothermal transfer.

Figure S.3. A correlation matrix showing R values for the core 32 VOCs in breath using the DB-SPME sampling method.

Figure S.4. A correlation matrix showing R values for the core 35 VOCs in breath using the cryothermal transfer sampling method.


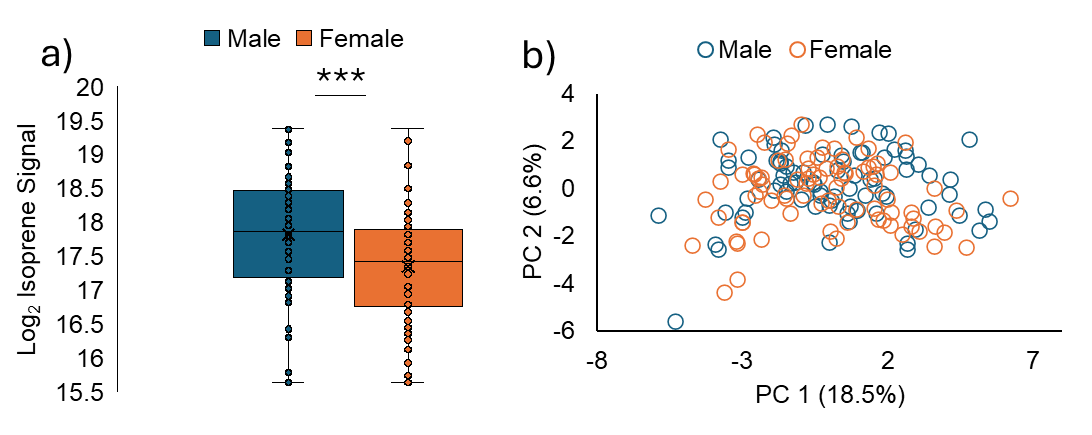


Figure S.5. Investigations into biological sex was performed within the DB-SPME sample cohort with (a) male volunteers displaying statistically significantly elevated isoprene levels relative to women (*p* value = 0.0006). (b) Multivariate analysis was also performed with PCA being unable to distinguish male from female volunteers.
